# Supplementary material for: Grade 3–4 Immune-Related Adverse Events Induced by Immune Checkpoint Inhibitors in Non-Small-Cell Lung Cancer (NSCLC) Patients Are Correlated with Better Outcome: A Real-Life Observational Study
Source: Cancers (Basel). 2022 Aug 11;14(16):3878. doi: 10.3390/cancers14163878 (PMC9405595; doi:10.3390/cancers14163878)
Supplement: Supplementary file 1 [file cancers-14-03878-s001.zip › cancers-1842914-supplementary-.pdf]

**Supplementary Figure S1:** TTNT in the entire population

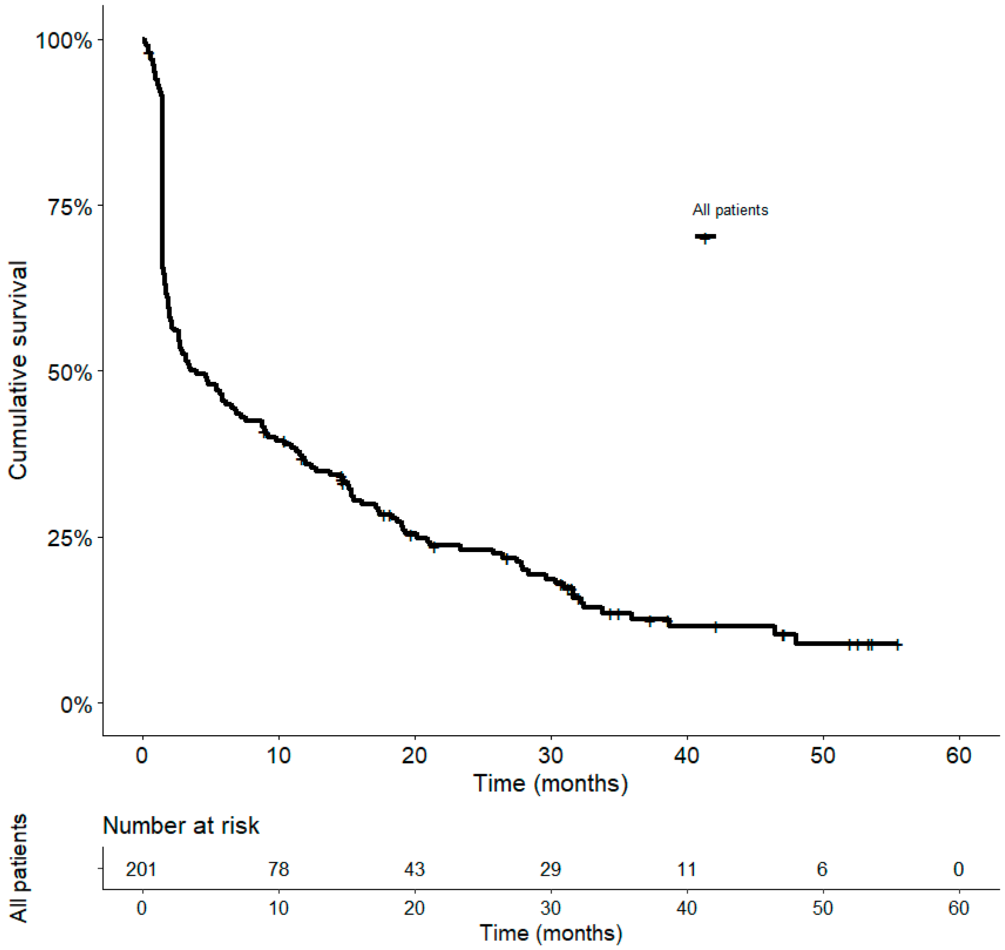

**Supplementary Figure S2:** TTNT according to treatment with antibiotics **(A),** corticosteroids **(B),** and LIPI score **(C).**

**Suppl. Fig. S2A:**

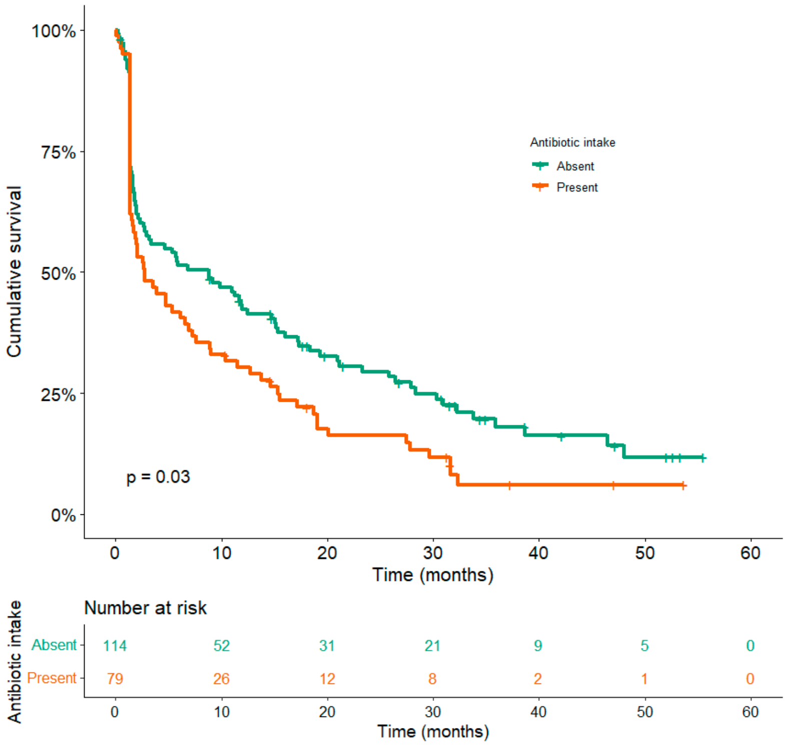


**Suppl. Fig. S2B:**

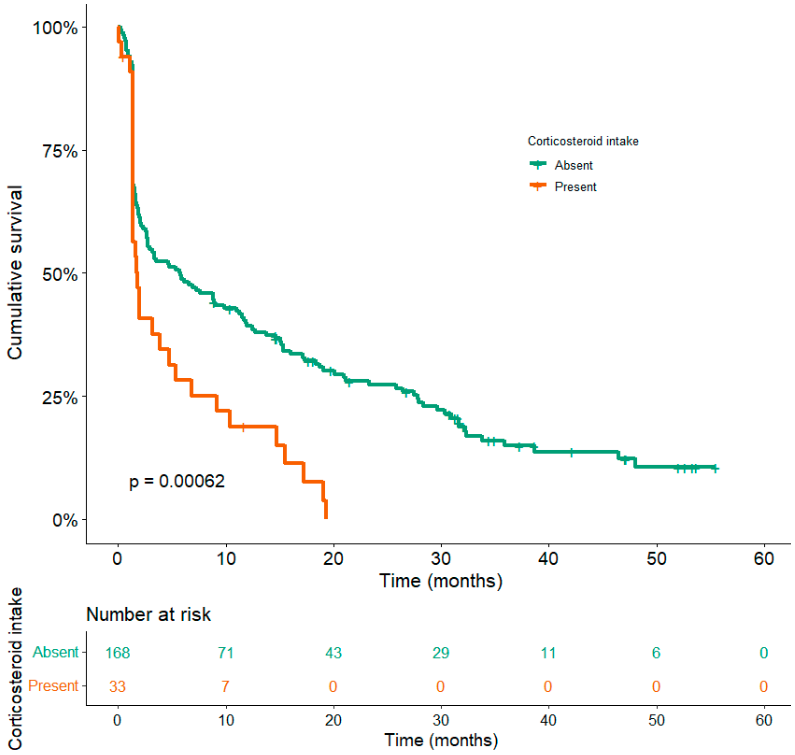

**Suppl. Fig. S2C:**

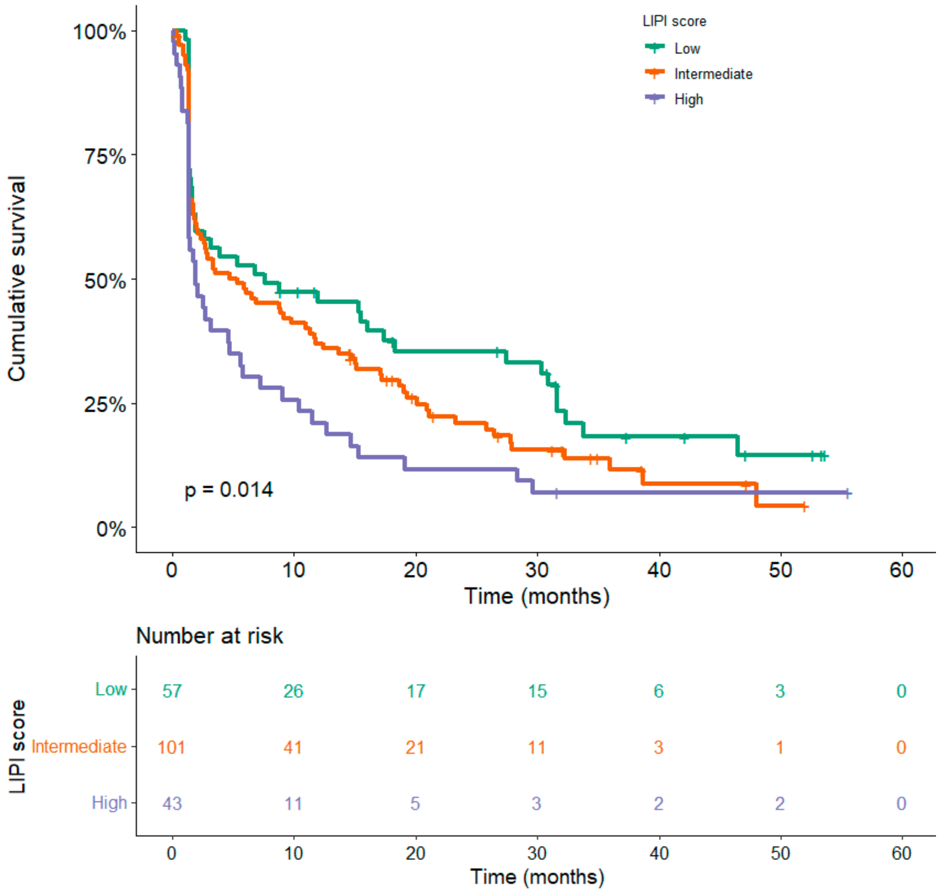

**Supplementary Table S1 -** Comparison of patients with Grade 3-4 immune-related adverse events with patients without Grade 3-4 irAEs

| **Variables** | **N** | **All patients (% of all patients)** | **N** | **Grade 3-4 irAEs** | | **p** |
|---|---|---|---|---|---|---|
| | | | | No (% of patients in each variable) | Yes (% of patients in each variable) | |
| **Age at ICI initiation (years)**<br>**Mean (SD)**<br>**Median (IQR)** | 201 | 63.3 (10.4)<br>64 (57 - 71) | 201 | 62.9 (SD: 10.7)<br>63 (IQR: 56-70.5) | 64.9 (SD: 9.4)<br>66.5 (IQR: 61-72) | 0.24 |
| **Gender**<br>**Male**<br>**Female** | 201 | 130 (65)<br>69 (35) | 201 | 110 (84.5.)<br>55 (80) | 22 (17)<br>14 (20.5) | 0.57 |
| **Healthcare center**<br>**Bichat – Claude Bernard AP-HP**<br>**S[t] Joseph Foundation Hospital** | 201 | 162 (80.5)<br>39 (19.5) | 201 | 131 (81)<br>34 (87) | 31 (19.5)<br>5 (13) | 0.37 |
| **Smoking status**<br>**Never-smokers**<br>**Former smokers**<br>**Smokers** | 201 | 11 (5)<br>90 (45)<br>100 (50) | 201 | 8 (73.0)<br>74 (82)<br>83 (83) | 3 (27.5)<br>16 (18)<br>17 (17) | 0.65 |
| **Histology**<br>**Squamous cell carcinoma**<br>**Non-squamous cell carcinoma** | 201 | 55 (27.5)<br>146 (72.5) | 201 | 48 (87.5)<br>117 (80) | 7 (13)<br>29 (20) | 0.30 |
| **Performance status**<br>**0**<br>**1**<br>**2**<br>**3** | 201 | 27 (13.5)<br>94 (47)<br>69 (34)<br>11 (5.5) | 199** | 21 (78)<br>76 (81)<br>57 (82.5)<br>11 (100) | 6 (22)<br>18 (19.5)<br>12 (17.5)<br>0 (0) | 0.45 |
| **Metastatic sites count**<br>**< 3**<br>**≥ 3** | 201 | 102 (51.0)<br>99 (49.0) | 199** | 83 (81.5)<br>82 (83) | 19 (19)<br>17 (17.5) | 0.85 |
| **Brain metastasis**<br>**No**<br>**Yes** | 201 | 136 (68.0)<br>65 (32.0) | 199** | 108 (79.5)<br>57 (88) | 28 (21)<br>8 (12.5) | 0.17 |
| **Liver metastasis**<br>**No**<br>**Yes** | 201 | 168 (84.0)<br>33 (16.0) | 199** | 134 (80)<br>31 (94) | 34 (20.5)<br>2 (6) | 0.08 |

| | | | | | | |
|---|---|---|---|---|---|---|
| **Disease stage at ICI initiation** | | | | | | |
| **Non metastatic** | 201 | 25 (12.5) | 199** | 19 (76) | 6 (24) | 0.41 |
| **Metastatic** | | 176 (87.5) | | 146 (83) | 30 (17) | |
| **History of CRD** | | | | | | |
| **No** | 192 | 131 (68.0) | 191# | 107 (82) | 24 (18.5) | 1.00 |
| **Yes** | | 61 (32.0) | | 50 (82) | 11 (18.5) | |
| **History of CVD** | | | | | | |
| **No** | 195 | 94 (48.2) | 194## | 82 (87) | 12 (13) | 0.06 |
| **Yes** | | 101 (51.8) | | 77 (76) | 24 (24) | |
| **PPI intake*** | | | | | | |
| **No** | 182 | 109 (59.9) | 181‡ | 94 (86) | 15 (14) | **0.034** |
| **Yes** | | 73 (40.1) | | 53 (72.5) | 20 (27.5) | |
| **Corticosteroids intake*** | | | | | | |
| **No** | 201 | 168 (83.6) | 199** | 136 (81) | 32 (19) | 0.46 |
| **Yes** | | 33 (16.4) | | 29 (88) | 4 (12.5) | |
| **Antibiotics intake*** | | | | | | |
| **No** | 193 | 114 (59.1) | 192‡‡ | 93 (81.5) | 21 (18.5) | 1.00 |
| **Yes** | | 79 (40.9) | | 64 (81) | 15 (19) | |
| **ICI sequence** | | | | | | |
| **First-line** | 201 | 61 (30.3) | 199** | 49 (80.5) | 12 (20) | 0.84 |
| **Following chemotherapy** | | 140 (69.7) | | 116 (83) | 24 (17.5) | |
| **ICI type** | | | | | | |
| **Nivolumab** | | 138 (68.7) | | 116 (84) | 22 (16) | **0.023** |
| **Pembrolizumab** | 201 | 51 (25.4) | 199** | 43 (84.5) | 8 (16) | |
| **Nivolumab + Ipilimumab** | | 12 (6.0) | | 6 (50) | 6 (50) | |
| **PD-L1 TPS** | | | | | | |
| **<1 %** | | 33 (20.9) | | 31 (94) | 2 (6) | 0.11 |
| **1-49 %** | 158 | 37 (23.4) | 156‡‡‡ | 30 (81) | 7 (20) | |
| **≥50 %** | | 88 (55.7) | | 68 (77.5) | 20 (23) | |

SD, standard deviation; IQR, interquartile range; irAEs, immune-related adverse events; ICI, immune checkpoint inhibitor; CRD, chronic respiratory disease; CVD, cardiovascular disease; PPI, proton pump inhibitor; TPS, tumor proportion score.

* during the month preceding and/or the three first months following the initiation of ICI treatment.

** missing data in 2 patients; # missing data in 10 patients; ## missing data in 7 patients; ‡ missing data in 20 patients

‡‡missing data in 9 patients; ‡‡‡missing data in 45 patients

**Supplementary Table S2 –** Univariable ~~(Kaplan-Meier)~~ and multivariable analyses (Cox proportional hazards) for time to new treatment (TTNT)

| | | | Univariable analysis | | | Multivariable analysis | |
|---|---|---|---|---|---|---|---|
| **Variables** | **N (events)** | **HR** | **95% CI** | **P value** | **aHR** | **95% CI** | **P value** |
| ***Age at ICI initiation (years)*** | 201 (169) | | | | | | |
| **> 65** | 91 (75) | - | | 0.286 | | | |
| **≤ 65** | 110 (94) | 1.2 | 0.9-1.6 | | | | |
| ***Gender*** | 201 (169) | | | | | | |
| **Female** | 69 (51) | - | | **0.018** | 1.5 | 1.1-2.1 | **0.020** |
| **Male** | 132 (118) | 1.5 | 1.1-2.1 | | | | |
| ***PS at ICI initiation*** | 201 (169) | | | | | | |
| **0-1** | 121 (94) | - | | **<0.0001** | 1.5 | 1.1-2.1 | **0.010** |
| **≥ 2** | 80 (75) | 1.9 | 1.4-2.6 | | | | |
| ***Antibiotic intake**** | 193 (161) | | | | | | |
| **No** | 114 (90) | - | | **0.039** | 1.4 | 1.0-1.9 | 0.062 |
| **Yes** | 79 (71) | 1.4 | 1.0-1.9 | | | | |
| ***Corticosteroid intake**** | 201 (169) | | | | | | |
| **No** | 168 (138) | - | | **0.002** | 1.6 | 1.0-2.4 | **0.034** |
| **Yes** | 33 (31) | 1.9 | 1.3-2.8 | | | | |
| ***Histological type*** | 201 (169) | | | | | | |
| **Squamous** | 55 (49) | - | | 0.597 | | | |
| **Non-squamous** | 146 (120) | 0.9 | 0.7-1.3 | | | | |
| ***Number of metastatic sites*** | 201 (169) | | | | | | |
| **< 3** | 102 (78) | - | | **<0.0001** | 1.6 | 1.1-2.2 | **0.017** |
| **≥ 3** | 99 (91) | 1.9 | 1.4-2.6 | | | | |
| ***PD-L1 status (TPS)*** | 158 (129) | | | 0.108 | | | |
| **≥ 50** | 88 (67) | - | | - | | | |
| **0** | 33 (31) | 1.5 | 1.0-2.4 | 0.048 | | | |
| **1-49** | 37 (31) | 1.3 | 0.9-2.0 | 0.189 | | | |
| ***Disease stage at ICI initiation*** | 201 (169) | | | | | | |
| **Non metastatic** | 25 (21) | - | | 0.133 | | | |
| **Metastatic** | 176 (148) | 1.4 | 0.9-2.3 | | | | |
| ***Brain metastasis*** | 201 (169) | | | | | | |
| **No** | 136 (107) | - | | **<0.001** | | | |
| **Yes** | 65 (62) | 1.8 | 1.3-2.5 | | | | |
| ***Liver metastasis*** | 201 (169) | | | | | | |
| **No** | 168 (137) | - | | **<0.0001** | 1.9 | 1.2-2.9 | **0.006** |
| **Yes** | 33 (32) | 2.4 | 1.6-3.5 | | | | |
| ***ICI sequence*** | 201 (169) | | | | | | |
| **First-line** | 61 (49) | - | | 0.828 | | | |
| **Following chemotherapy** | 140 (120) | 1.0 | 0.7-1.4 | | | | |
| ***ICI type*** | 201 (169) | | | | | | |
| **Nivolumab and Ipilimumab** | 12 (10) | - | | 0.328 | | | |
| **Nivolumab or Pembrolizumab** | 189 (159) | 1.4 | 0.7-2.6 | | | | |
| ***LIPI score*** | 201 (169) | | | **0.022** | | | **0.023** |
| **0** | 57 (44) | - | | - | - | | - |
| **1** | 101 (85) | 1.3 | 0.9-1.9 | 0.155 | 1.7 | 1.1-2.5 | **0.010** |
| **2** | 43 (40) | 1.8 | 1.2-2.8 | **0.006** | 1.7 | 1.1-2.7 | **0.023** |
| ***Grade 3-4 irAEs*** | 201 (169) | | | | | | |
| **Yes** | 36 (25) | - | | **<0.0001** | 2.4 | 1.6-3.8 | **<0.0001** |
| **No** | 165 (144) | 2.4 | 1.6-3.7 | | | | |

* During the month preceding and/or the three first months following the initiation of ICI treatment. 95% CI, 95% confidence interval; aHR, adjusted hazard ratio; HR, hazard ratio; ICI, immune checkpoint inhibitor; irAEs, immune-related adverse events; PD-L1, programmed death ligand 1; PS, performance status, TPS, tumor proportion score; TTNT, time to new treatment.

The multivariable analysis included 193 patients with all available data, accounting for 161 events.

**Supplementary Table S3 –** Multivariable analysis by Cox proportional hazards for overall survival (OS), including PD-L1

| **Multivariable analysis** (events = 102/151) | | | |
|---|---|---|---|
| **Variables** | **aHR** | **95% CI** | **P value** |
| ***Gender*** | | | |
| Female | 1.8 | 1.1-2.8 | **0.014** |
| Male | | | |
| ***PS at ICI initiation*** | | | |
| 0-1 | 1.9 | 1.2-2.9 | **0.005** |
| ≥ 2 | | | |
| ***Antibiotic intake**** | | | |
| No | 1.7 | 1.1-2.6 | **0.017** |
| Yes | | | |
| ***Corticosteroid intake**** | | | |
| No | 2.2 | 1.2-3.9 | **0.010** |
| Yes | | | |
| ***Number of metastatic sites*** | | | |
| < 3 | 1.6 | 0.9-2.6 | 0.088 |
| ≥ 3 | | | |
| ***Brain metastasis*** | | | |
| No | 1.7 | 1.0-2.9 | **0.035** |
| Yes | | | |
| ***Liver metastasis*** | | | |
| No | 2.5 | 1.4-4.2 | **0.001** |
| Yes | | | |
| ***LIPI score*** | | | **<0.001** |
| 0 | - | | **-** |
| 1 | 3.2 | 1.8-5.6 | **<0.0001** |
| 2 | 2.2 | 1.2-4.0 | **0.007** |
| ***Grade 3-4 irAEs*** | | | |
| No | 3.4 | 1.9-6.4 | **<0.0001** |
| Yes | | | |

* During the month preceding and/or the three first months following the initiation of ICI treatment.
95% CI, 95% confidence interval; aHR, adjusted hazard ratio; ICI, immune checkpoint inhibitor; irAEs, immune-related adverse events; PS, performance status.

PD-L1 was included in the modeling procedure, the multivariable analysis including 151 patients with all available data accounting for 102 events. PD-L1 is not retained in the final model. Stage was excluded either from the model since tightly linked to brain, liver and number of metastases variables.
